# Evaluation of an educational, theater-based intervention on attitudes toward organ donation in Risaralda, Colombia

**Published:** 2013-03-30

**Authors:** Juliana Buitrago, Sandra Gómez, Alvaro Guerra, Leidy Lucumí, César Romero, Julio Sánchez

**Affiliations:** a Health Sciences Faculty, Universidad Tecnológica de Pereira, Pereira, Colombia, E-mails: jcsanchez@utp.edu.co; b Organization for Organ Procurement, (O. P. O.) Colombia Vive, Pereira, Colombia, E-mail: tatagomez27@hotmail.com

**Keywords:** Education, tissue and organ procurement, drama, organ transplantation, healtcare acceptability, organ donations

## Abstract

**Introduction:**

The shortage of organs for transplantation is a worldwide problem and the main cause is the refusal of family members to donate. Consent to donate is influenced by many factors and educational interventions are strongly recommended.

**Objective:**

To evaluate the impact of an educational, theaterbased strategy on the attitudes toward organ donation.

**Methods:**

This study employed an intervention using theater as the central tool. The impact of this intervention on the intention to donate was assessed through a controlled, prospective, nonrandomized designed study. The sample consisted of 1,038 people. All the participants answered a survey that asked about sex, age and intent to donate. Afterward, one portion of the sample was exposed to the play, The Gift of Life, and a subsequent discussion forum that was guided by experts. The same survey was administered again after the intervention.

**Results:**

Before the intervention, donation attitudes were positive in 68.3% of the responses, negative in 6.8% and uncertain in 24.9%. Females showed a greater intent to donate while age had no apparent influence on the donation decision. Those exposed to the intervention were found to be more likely to donate and show a favorable change in attitude toward donation than those who were not exposed to the intervention.

**Conclusion:**

An educational intervention using theater is an effective tool to generate a short-term change in the intent to donate. Educational strategies should be employed to increase the rates of organ donation.

## Abstract (es)

**Introducción:**

La escasa disponibilidad de órganos para transplantes es un problema de escala mundial y la principal causa es la negativa de los familiares del donante. El consentimiento para donar está influenciado por una multitud de factores y las intervenciones educativas han sido intensamente recomendadas.

**Objetivo:**

Evaluar el impacto de una estrategia pedagógica basada en el teatro sobre las actitudes hacia la donación de órganos.

**Metodología:**

Este estudio utilizó una intervención teatral como herramienta central de trabajo y evaluó su impacto sobre la intención de donación usando un diseño controlado, prospectivo y no aleatorizado. La muestra consistió de 1,038 personas. Todos los participantes respondieron una encuesta que preguntaba acerca del género, edad e intención de donación. Después de ésta, una parte de la muestra fue expuesta a la obra teatral El Regalo de la Vida, seguida de un foro de discusión con expertos. La misma encuesta inicial fue aplicada de nuevo después de la intervención.

**Resultados:**

Antes de la intervención, las actitudes hacia la donación fueron positivas en 68.3% de los casos, negativa en 6.8% e incierta en 24.9%. Las mujeres mostraron una mayor intención de donación mientras que la edad no tuvo ninguna influencia. Aquellas personas expuestas a la intervención estuvieron más inclinadas hacia la donación y mostraron un cambio favorable en su actitud que aquellas que no fueron expuestas a ésta.

**Conclusión:**

Una intervención educativa usando el teatro es una herramienta efectiva para generar un cambio en al intención de donación de órganos. Estrategias educativas similares a ésta deben implementarse para incrementar la tasa de donación de órganos entre la población general.

## Introduction

One of the most important medical advances in the last fifty years involves solid organ transplantation. It is the best therapeutic alternative for terminal organ failure and in many cases it is the only alternative to death. However, a shortage of organs available for transplants exists and this shortage should be considered a public health problem^1^. In Colombia, for instance, there are 1,007 patients on the waiting list for an organ^2^. However, because of the shortage of available organs, some of these patients will die. In Risaralda, a densely populated region in the center of Colombia, the problem is significant. According to the number of dialysis units in the area, there are approximately 500 terminal renal patients. These patients represent a high economic and social burden. This problem is exacerbated by an increasingly deficient donation culture, which leads to the need for developing strategies to increase donation rates^3^.

The possibility of obtaining an organ in Colombia, where more than 90% of organs come from deceased donors, depends on the authorization of families^2^. This altruistic act is greatly influenced by a solid comprehension of the donation and transplantation process^4^. A family's consent to donate an organ depends on multiple factors, many of which have been identified in different studies around the world^5^ ^,^ ^6^. One of the most important factors is the will of the deceased regarding donation as expressed during their lifetime ^7^. The decision to deny a donation is often the result of not knowing the deceased's will, or there being disagreement among family members^8^ ^,^ ^9^. It is therefore very important that people consider the possibility of donation while they are alive and clearly express their will to their families. Because the clear expression of will is critical for increasing the rate of organ donation, it is necessary to raise the public's consciousness of this issue^10^. A community educational intervention could improve donation rates in the long term by clarifying doubts, discussing myths and promoting altruistic behavior^11^. Discussion about the concept of cerebral death and contact with successful recipients may transform skeptics into believers.

Traditional interventions based on lectures, talks and informative brochures neither significantly influence the community conscience nor generate a process of awareness, internalization or personal reflection that might lead to a change in the attitude and behavior concerning this subject. Furthermore, traditional interventions show a significant loss of retention over periods as short as three months ^12^.

In Colombia, the National Transplant Net demonstrated that promotion of donation leads to an increased transplant quantity^13^. The didactic strategies used to deliver this information must create interpersonal discussions that generate interest among the audience. These strategies must encourage the audience to view themselves as protagonists in the situation. In other words, the viewers should feel personally involved in the situation, be able to relate to the experience, and engage their cognitive, affective and behavioral faculties. Strategies such as these are necessary to produce significant change in attitudes. They are the only ways in which the audience can visualize themselves as possible donors and become active and critical advocates who promote organ donation in their communities.

Theater is an effective pedagogical tool that has been found to be useful in approaching a diversity of health subjects, especially ethical issues^14^. Although the effectiveness of theater is supported by a number of studies ^15^ ^,^ ^16^, there are no reported studies directly related to the culture of organ donation.

Theater is a unique tool because it facilitates the reproduction of different situations, but the challenge with theater is that it uses a special language that emphasizes emotive communication. In addition, theater stimulates creativity and enjoyment while being able to transmit a message in a way that does not alienate the spectator. These characteristics are in contrast to those of other communicative strategies.

##  Materials and Methods 

This was a controlled, prospective, non-randomized study that employed a theater-based strategy to deliver information about organ donation and transplants to a community in Risaralda, Colombia. The study was approved by the Ethics Committee of the Technological University of Pereira, Colombia.

The sample included 1,038 people from 8 Risaralda municipalities (separate from the capital). None of the participants worked in the health sector, so that their knowledge of the subject was likely only basic or, as in many cases, non-existent. The sample included people who freely accepted the invitation to participate in the experiment after encouragement to attend by community leaders, as well as exposure to related publicity. The sample was not selected by age, gender, sexual orientation, religious beliefs, educational level or any other parameter.

Prior to the interventions, research was conducted to identify the principal myths and key points to address. The latter included the following items: ignorance about the will of the deceased, disfigurement of the corpse, inaccurate beliefs about cerebral death, ignorance about the organ assignment process, organ trafficking, fear about the removal of organs before death, inadequate medical attention to potential donors and worries about the costs of the process. All of these subjects were considered in designing the intervention. Other important topics that were addressed included: the role of procurement and transplant coordinators, informed consent, autonomy of the family in making the donation decision, aspects of the family interview, anonymity, waiting list procedures, the possibility of being a recipient after being a donor or authorizing a donation, the possibility of passing away before receiving an organ, personal and family uncertainty while someone is on a waiting list, the importance of expressing one's own will about organ donation during one's lifetime, satisfaction after the altruistic act of donation, its impact on mourning and the way in which donation may influence the life of the people involved.

The core of the pedagogical intervention was a play staged especially for this study by The Diving Bell Theater Group (La Escafandra Teatro). This group has recognized experience using theater as a tool for educating people about health topics^15^ and it was guided by a team of experts from the Colombia Vive Organ Procurement Organization (O.P.O.). The experts included two transplant surgeons and the regional transplant coordinator. The twenty minute play, named The Gift of Life, portrayed all the possible roles a person can assume in the process of transplantation, such as donor, recipient, patient on a waiting list, family member and health worker. These roles demonstrated the different viewpoints about the issue and the possible ways in which people can perceive organ donation and transplants. The play also raised the most common myths and beliefs about this topic through the story of Arturo, a young man who suffered an accident with resulting cerebral death. His mother, who was initially reluctant to authorize the organ donation, was eventually convinced by her daughter to go through with it. The mother needed a cornea transplant a few years later and reflection about her experience leads her to the logical conclusion that any person may need a donor at some point in life. Prior to the intervention, the team of experts visited the municipalities to meet community leaders, hospital managers, school principals and others identified as possessing the potential to orBuitrago ganize meetings and summon their respective community leaders. The team explored the available resources for adapting the play, while clearly explaining its methodology and objectives toward overcoming initial barriers. The intervention was programmed according to the suggestions made by these leaders to optimize attendance. The particular circumstances of each community were considered.

The intervention started with a presentation by the team of experts and artists, followed by an explanation about the ethical aspects of the research. All of those in attendance signed an informed consent to participate in the study. Afterward, an anonymous survey with three questions was administered to the audience members. The survey asked about gender (male, female), age (in years) and intention to donate with the question, "Would you donate your organs after your death?" (yes, no, I do not know/I do not respond). Long-time members of the team were available to help people, especially illiterate people, to understand the survey.

After the survey was completed, the play was presented to the audience members. Afterward, a discussion forum was conducted that allowed open participation and unlimited time for responses. This forum included the participation of one or two members from the Capullos Foundation, an association of transplant patients. The members provided testimonials about their experiences. All of the questions from the audience were answered by the members of the expert committee. Before the activity ended, the same survey was once again administered. A second group was composed of people who were not exposed to the play or the discussion forum. The same survey was administered to both groups. STATA 8.0 software was employed to analyze the data. Univariate and bivariate analyses were conducted and a *p* <0.05 significance level was used. Additionally, Chi Square and Mantel-Hazel tests were applied to determine whether the probability of an event, in this case organ donation intention, was the same for the two groups.

## Results

In all of the municipalities, there was a willingness to consider organ donation and participate in the experiment. All of the participants responded to the survey, attended the play and participated in the final discussion which demonstrated interest in the activity. In the sample, 58% of the participants were female, ranging in age from 10 to 85 years (mean 24 ± 14.5 years). Sixty-one percent were less than 18 years old, which is considered the adult age in Colombia and in the majority of occidental countries. 68.3% declared that they would donate organs in the case of death, 6.8% declared they would not donate and 24.9% marked the option I do not know/I do not respond. Women showed a significantly greater intention to donate in comparison with men (*p*=0.000). In contrast, there were no significant differences between the age groups concerning the intention to donate, and no differences were found between people older than 18 years and those younger than 18 years (Table 1).

**Table 1:**
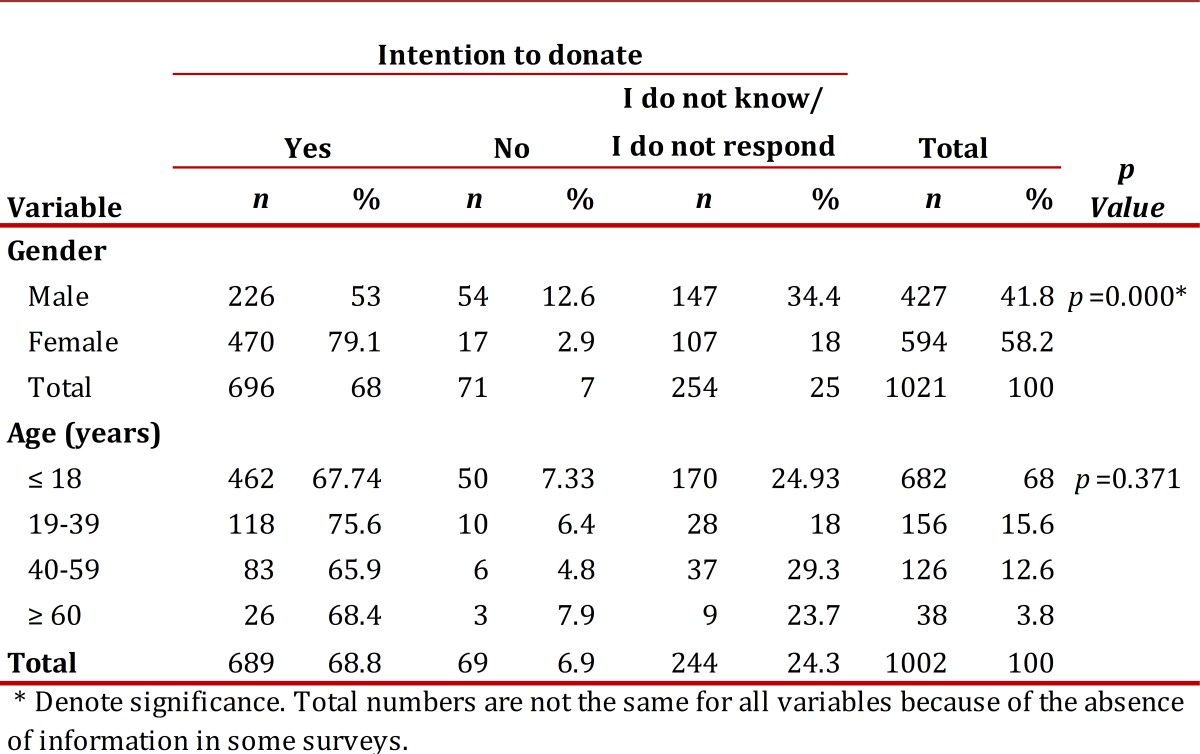
Evaluation of the intention to donate organs and tissues after death in people non-exposed to the intervention according with the variables analyzed

**Figure 1:**
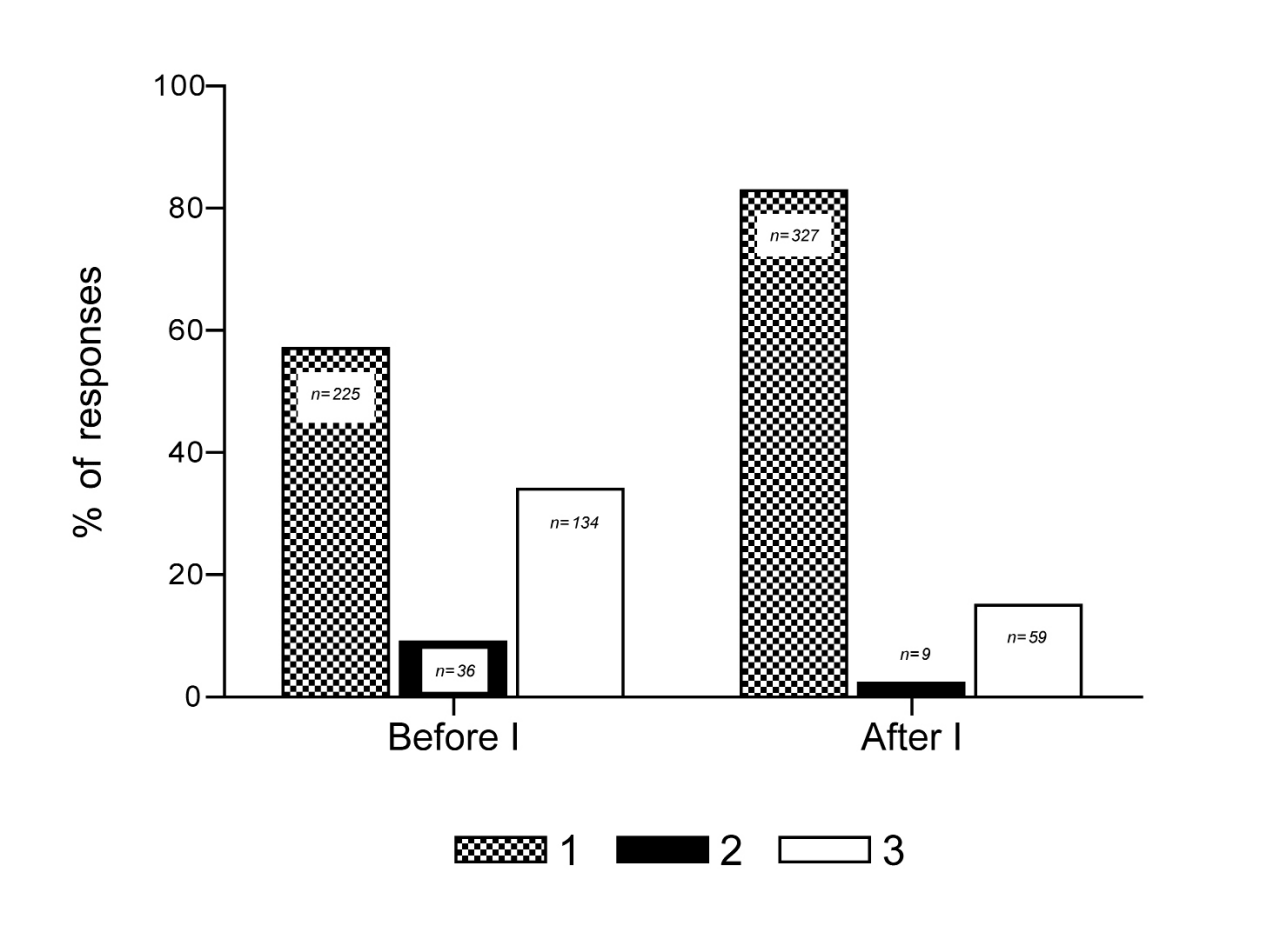
Comparison between the responses to the question "Would you donate your organs after you have died?" before and after the intervention organs after you have died?" before and after the intervention (I).1. yes 2. No 3. I do not know/I do not respond

Among those in the sample, 43.2% were exposed to the intervention. Those exposed were selected at random from all of the participating municipalities and had a similar distribution by age and gender. The intention to donate organs in this sample changed significantly after the intervention: 74.24% of the people who declared that they would not donate and 55% of the people who were indecisive changed their opinion in favor of organ donation after the intervention (Fig. 1). The difference between the proportions of people with the intention to donate before and after the intervention was significant (*p* < 0.05).

## Discussion

The program was well-received by all of the municipalities involved, which was thought to be attributable to the preliminary visits. There were cultural barriers discovered, such as false beliefs about the involvement of satanic sects in organ donation, fear of being placed on donor lists without authorization, and fear of discussing death. These issues and others were addressed in the discussion forum after the play. The presence of patients who had undergone a previous organ transplant in each session was reported as helpful for people to understand better the play and more of the situational issues concerning transplants.

Despite the importance of religious beliefs about the intention to donate, as reported by others, the issue was not expressed as a significant one during the discussions. None of the participants declared that they were opposed to transplants because of religious reasons. Catholicism, which approves organ transplants ^17^, is the main religion in this country. The play, The Gift of Life, had an excellent reception. Afterwards, the audience congratulated the artists and expressed their satisfaction with the play. It was described as "emotive", "beautiful", "excellent", "shocking" and more. This positive reception was another demonstration of the positive effect the play had on the audience.

Regarding the intention to donate, the finding of this study (68.3% positive) is in accord with other research ^18^; however, it is lower than that reported by others ^19^. In the analysis of the age groups, this study could not find differences regarding the intention to donate, which is in contrast with other research ^18^ ^,^ ^20^. The greater intention to donate among women was remarkable. This finding, however, is consistent with a previous study involving a Spanish sample^9^.

Schauenburg and Hildebrandt studied differences in knowledge about transplants between German and Spanish populations ^21^. Their hypothesis was that a difference in knowledge explains the different rates in organ donation between the two countries. The study showed, however, that there was no difference in transplant knowledge between these two populations ^21^. This finding suggests that organ donation is a process that involves much more than knowledge about the topic. It also involves emotions, feelings, thoughts, beliefs and personal experiences ^10^. Theater can be useful for sensitizing people to the subject because it expresses all aspects of the issue and emotional reactions on the stage. The play The Gift of Life managed to communicate the main issues identified in other studies, such as comprehension about cerebral death,discussion of the topic in the family group, attitudes of parents toward donation, attitudes toward cremation, attitudes toward necropsy, fear about not being properly treated and concern about mutilation after donation^19^ ^,^ ^22^. All of these elements were incorporated into the play and were discussed in the forum in such a way that people could clarify their doubts and myths. The positive factors about transplants, such as the resultant altruistic feelings and pride in being a donor, were also reinforced by the play and the subsequent discussion.

The results of this study show that a theater-based intervention can change the expressed intention to donate organs among people exposed to it. All of the studies agree on the importance of education in organ donation^23^, since education has been identified as a key method for increasing organ availability.

Our strategy effectively increased the intention to be a donor, which indicates that it is possible to modify the intention to donate organs using a pedagogical strategy. However, the design of this study did not allow for an evaluation of the impact of the intervention in the actual decision making situation of potential donation. Further research about this question should be conducted with this objective, perhaps by following these people over a considerable period of time to re-assess the retention of the attitudinal change.

This study has a number of limitations; first, the study population was predominantly juvenile, which reduces any generalization of the results to other age groups; second, this study was conducted in a country with a specific religious, cultural and educational context which constitutes a very important conditioning for the intention to donate and limits the extension of the results. The educational efforts directed to the promotion of positive attitudes toward donation are a key way to increase donation rates ^11^ ^,^ ^24^ ^,^ ^25^, and the design of tailored, contextual approaches must be increased and enhanced to improve the process and desired outcomes.

In conclusion, there are two important findings from this study: (1) theater is an effective tool for modifying attitudes toward organ donation, and (2) educational interventions must be undertaken at the community level since this is the only way in which a real organ donation culture can be constructed.

## References

[1] Cantarovich F (2002). The organ shortagea social paradox to be reversed. Transplant Proc.

[2] INS, Instituto Nacional de Salud C (2010). Informe final Red Nacional de Donación y Trasplantes.

[3] Whiting JF, Kiberd B, Kalo Z, Keown P, Roels L, Kjerulf M (2004). Cost-effectiveness of organ donationevaluating investment into donor action and other donor initiatives. Am J Transplant.

[4] Abouna GM (2003). Ethical issues in organ transplantation. Med Princ Pract.

[5] Alvaro EM, Jones SP, Robles AS, Siegel JT (2005). Predictors of organ donation behavior among Hispanic Americans. Prog Transplant.

[6] Anker AE, Feeley TH (2011). Asking the Difficult QuestionsMessage Strategies Used by Organ Procurement Coordinators in Requesting Familial Consent to Organ Donation. J Health Commun.

[7] Siminoff LA, Gordon N, Hewlett J, Arnold RM (2001). Factors influencing families' consent for donation of solid organs for transplantation. JAMA.

[8] Martinez JM, Lopez JS, Martin A, Martin MJ, Scandroglio B, Martin JM (2001). Organ donation and family decision-making within the Spanish donation system. Soc Sci Med.

[9] Rando Calvo B., Blanca M. J., de Frutos MA (2002). La toma de decisión sobre donación de órganos en la población andaluza. Psicothema.

[10] Schulz PJ, Nakamoto K, Brinberg D, Haes J (2006). More than nation and knowledge: cultural micro-diversity and organ donation in Switzerland. Patient Educ Couns.

[11] Cantarovich F (2002). Improvement in organ shortage through education. Transplantation.

[12] González RP, JM, Jiménez O, Rodríguez MS, Vásquez P, González F (2001). Familiares de pacientes en muerte encefálica: actitudes frente a la muerte y donación de órganos / Patient´s family in brain death: attitudes in front of death and organ donation. Boletín del Hospital de San Juan de Dios.

[13] Bohorquez H (1999). Red de trasplantesun trabajo en equipo para Santafé de Bogotá / The organ transplantation network: teamwork for Santafé de Bogotá. Rev. Colomb. Cir.

[14] Bartlett R, Townsend N (1998). Using drama to promote discussion of ethical issuesPig in the Middle. Bull Med Ethics.

[15] Sanchez JC, Gutierrez JC, Morales MD (2010). Cinema and theater as training tools for health students. Fam Med.

[16] Seguin A, Rancourt C (1996). The theatrean effective tool for health promotion. World Health Forum.

[17] Conesa Bernal C, Rios Zambudio A, Ramirez Romero P, Parrilla Paricio P (2004). The Catholics and organ donations. Med Clin.

[18] Yeung I, Kong SH, Lee J (2000). Attitudes towards organ donation in Hong Kong. Soc Sci Med.

[19] Rios A, Cascales P, Martinez L, Ramirez P, Sanchez J, Jarvis N (2008). Attitude of Scottish residents living in southeastern Spain toward organ donation. Transplant Proc.

[20] Colak M, Ersoy K, Haberal M, Gurdamar D, Gercek O (2008). A household study to determine attitudes and beliefs related to organ transplantation and donationa pilot study in Yapracik Village, Ankara, Turkey. Transplant Proc.

[21] Schauenburg H, Hildebrandt A (2006). Public knowledge and attitudes on organ donation do not differ in Germany and Spain. Transplant Proc.

[22] Rios A, Febrero B, Martinez-Alarcon L, Lopez-Navas A, Sanchez J, Guzman D (2010). Evaluation of attitudes toward living organ donationa multicenter study of compulsory secondary school education teachers. Transplant Proc..

[23] Callender CO, Miles PV (2010). Minority organ donationthe power of an educated community. J Am Coll Surg.

[24] Cantarovich F (2002). Education, a chance to modify organ shortagea different message to society. Transplant Proc.

[25] Cantarovich F (2004). The role of education in increasing organ donation. Ann Transplant.

